# The Link Between Syntactic Complexity and Stuttering‐Like Disfluencies in French Speaking Adults

**DOI:** 10.1111/1460-6984.70218

**Published:** 2026-03-05

**Authors:** Alice Le Dévic, Sascha Diwersy, Ivana Didirková

**Affiliations:** ^1^ Institut des Sciences et Techniques de la Réadapatation, Université Claude Bernard Lyon 1 Lyon France; ^2^ UMR5267 Praxiling, Université de Montpellier Paul‐Valéry & CNRS Montpellier France

**Keywords:** adults who stutter, disfluency, IPSyn, ISC, MLU, syntactic complexity

## Abstract

**Background:**

Stuttering is a neurodevelopmental disorder characterized by speech disfluencies. While studies have shown a link between syntactic complexity and stuttering in children, its role in adults who stutter (AWS) remains unclear. This study investigates whether increased syntactic complexity correlates with stuttering‐like disfluencies (SLDs) in AWS and evaluates three syntactic complexity indices.

**Methods:**

Participants included 24 French‐speaking adults (12 AWS, 12 controls), matched for age, gender, and education. Participants completed a sentence description task using sentences of varying syntactic complexity. Syntactic complexity was quantified using three measures: Mean Length of Utterance (MLU), Index of Syntactic Complexity (ISC), and Index of Productive Syntax (IPSyn). Disfluencies were categorized as SLDs or other disfluencies (ODs). Statistical analyses examined the relationship between syntactic complexity and disfluencies across participant groups.

**Results:**

AWS produced significantly more SLDs than controls across all levels of syntactic complexity. The number of disfluencies increased with syntactic complexity in AWS but not in controls. MLU was the most sensitive index for predicting disfluencies. Severe stutterers exhibited higher disfluency rates compared to mild and moderate stutterers.

**Conclusion:**

Increased syntactic complexity exacerbates stuttering in AWS. MLU, a measure of sentence length, proved most effective for explaining disfluency rates, suggesting its clinical utility. Findings support the demands and capacities model, indicating that reducing syntactic demands may alleviate stuttering.

**WHAT THIS PAPER ADDS:**

*What is already known on this subject*

- Persistent developmental stuttering is characterized, among others, by an increased number of disfluencies, known as stuttering‐like disfluencies. Many linguistic factors influence the apparition of stuttering‐like disfluencies, such as phonological characteristics of disfluent sounds. Regarding morphosyntactic factors, it is known that disfluencies switch from function to content words in adults and studies on some languages show that syntactic complexity does influence stuttering in the adult population. However, such studies use different measures of syntactic complexity and are scarce for French.

*What this paper adds to existing knowledge*

- In our study, we tested a protocol designed to explore syntactic complexity and its relationship with stuttering. Disfluencies were elicited during speech production across varying levels of complexity, and three different measures were used to address the research question. Among these, MLU proved to be the most effective in explaining the occurrence of stuttering‐like disfluencies, confirming that sentence length plays a more significant role than sentence structure in adults who stutter.

*What are the potential or actual clinical implications of this work?*

- This study highlights the importance of syntactic complexity in stuttering, offering insights for clinical speech therapy and research. While stuttering therapy often focuses on children, these findings suggest that simplifying sentence production could also benefit adults who stutter. Speech therapists could work on improving the linguistic abilities of PWS, particularly syntax, to reduce disfluencies and build a stronger language foundation. The study also validates MLU as a reliable tool for measuring disfluencies, easily applicable in clinical practice. Combining these insights with existing programs, such as the Camperdown program, could enhance treatment by addressing both speech fluency and disorder awareness.

## 1 Introduction

Stuttering is a neurodevelopmental disorder defined by the Diagnostic and Statistical Manual of Mental Disorders V (APA 2013) as a “childhood‐onset fluency disorder.” It affects approximately 1% of the world's population — around 70 million people who stutter (PWS) — and occurs across all languages and socio‐cultural groups (Guitar 2019). In France, stuttering affects an estimated 650,000 individuals (Vincent 2013). The severity, frequency, and emotional impact of stuttering vary widely, and although the disorder resolves spontaneously or with speech therapy in around 80% of cases, it can persist into adulthood. Stuttering typically emerges between the ages of two and five, a period when language becomes increasingly complex (Guitar 2019). A substantial body of research has documented a link between linguistic demands, particularly syntactic complexity, and the frequency of stuttering in children. Whether this relationship persists into adulthood remains less well understood.

While existing literature highlights the effect of utterance length on stuttering in adults (Kleinow and Smith 2000), it remains unclear whether syntactic complexity is a significant predictor of stuttering‐like disfluencies (SLDs). To address this question, our study compares the number and types of disfluencies produced as a function of sentence length and syntactic complexity in French‐speaking adults who stutter (AWS) and in fluent control speakers.

Since the calculation of syntactic complexity must consider both utterance length and the degree of subordination (Hunt 1965), we employ three computational indices: the Mean Length of Utterance (MLU; Zackheim and Conture 2003) to estimate utterance length, and the Index of Syntactic Complexity (ISC; Szmrecsanyi 2004) and the Index of Productive Syntax (IPSyn; Scarborough 1990) to measure syntactic complexity per se.

## 2 Stuttering

Stuttering is a speech fluency disorder characterized by disruptions in speech fluency and rhythm. These disruptions encompass both **core behaviors** (referred to as SLDs) and **secondary behaviors**. SLDs manifest as repetitions of sounds, syllables, or short words, prolongations, and silent blocks, and are often accompanied by muscular tension (APA 2013). Secondary behaviors include involuntary body movements, loss of eye contact, inappropriate speech pauses, and emotional or physiological reactions such as anxiety, guilt, frustration, or laryngeal tension (Guitar 2019). Many PWS develop strategies to avoid anticipated difficulties, modifying the form, length, or structure of an utterance or even abandoning an utterance altogether to reduce the likelihood of stuttering (Junuzovic‐Zunic and Ibrahimagic 2013; Jackson et al. 2015). In addition, as suggested by Manning (2009), syntactic‐type disfluencies (e.g., revisions) tend to increase with age, while phonological disfluencies (e.g., repetitions or prolongations) may decrease, reflecting changes in linguistic processing and behavioral adaptation.

Linguistic factors, and syntactic factors in particular, have long been recognized as important in the manifestation of stuttering, especially in children. Disfluencies can involve phrase repetitions, syntactic breaks, or utterance reformulations (Van Borsel 1999). In children, stuttering often coincides with the acquisition of more complex syntactic structures, suggesting a bidirectional relationship between syntax and disfluencies: increased syntactic demands can trigger disfluencies, and disfluencies can in turn affect syntactic planning, at least during early language development (Guitar 2019). However, most of this research has been conducted in English, making it difficult to generalize findings to languages with different syntactic constraints, such as French (Kail 2000). Moreover, the methods used to operationalize syntactic complexity vary considerably, from measures of sentence length (e.g., number of syllables or morphemes using the MLU; Zackheim and Conture 2003) to indices of phrase composition (e.g., Developmental Sentence Scoring, DSS; Buhr and Zebrowski, 2009). While many studies report increased disfluency rates with greater syntactic complexity, others fail to find significant results, potentially due to methodological limitations. These include relying solely on subjective assessments by PWS, or using indices that fail to fully capture sentence structure, such as the MLU which does not account for syntactic complexity per se (Knijff et al. 2018); yet, following Hunt (1965), both length and subordination should be considered when quantifying complexity.

Although the link between syntactic complexity and stuttering has been extensively studied in children, less is known about its role in adults. As syntax becomes fully mastered during adolescence (Martinot 2013), its impact on fluency may shift. Children often use slower speech and increased pausing to manage linguistic load, whereas adults rely on more automatized syntactic processes. Consequently, AWS may display different patterns of syntax‐related disfluencies. Longitudinal studies further highlight variability in this relationship. For instance, Wagovich and Hall (2018) observed increased disfluency rates with longer utterances in children but found these patterns were not stable across time. This underlines the need to examine AWS directly rather than extrapolating from developmental data, as stuttering behaviors may evolve with age (Liu et al. 2018). Indeed, age‐related differences in lexical distribution suggest developmental changes in the interplay between language structure and stuttering. Children tend to stutter more on function words, whereas adults stutter more on content words (Au‐Yeung et al. 1998, Au‐Yeung et al. 2003). This shift may reflect differences linked to lexical frequency and planning strategies as language matures. Additionally, increasing syntactic complexity may compromise the stability of supraglottic articulatory movements in adults (Kleinow and Smith 2000), further linking linguistic structure and motor execution.

Overall, linguistic factors influencing stuttering are multifaceted, involving syntax, phonology, and lexical characteristics. Long and complex sentences can disrupt fluency in both PWS and fluent speakers (Ratner and Sih 1987), but their impact may be qualitatively different in PWS, potentially reflecting both increased processing demands and avoidance of complex structures. The present study aims to clarify this relationship by examining how sentence length and syntactic complexity relate to disfluency frequency and type in French‐speaking AWS compared with fluent controls. By combining objective measures of syntactic complexity with a focus on persistent developmental stuttering, this work seeks to shed light on how linguistic planning and speech motor control interact in adulthood. We use three complementary indices: MLU to estimate utterance length, and the Index of Syntactic Complexity (ISC; Szmrecsanyi 2004) and Index of Productive Syntax (IPSyn; Scarborough 1990) to capture structural complexity.

## 3 Measures of Syntactic Complexity

Syntax refers to the linguistic system through which words are combined to form meaningful sentences (Junuzovic‐Zunic and Ibrahimagic 2013). Accurately capturing syntactic complexity is crucial when investigating linguistic factors that may contribute to speech fluency breakdowns. Sentence complexity can vary considerably depending on its internal structure, and several indices have been developed to quantify these variations.

Among them, the MLU is one of the most widely used measures, particularly in studies on stuttering. It provides a robust estimate of utterance length based on morpheme counts (Frizelle et al. 2018). However, MLU alone does not adequately reflect syntactic complexity: an utterance can be long without being structurally complex (e.g., through concatenation), whereas a short utterance can be highly complex if it involves a high degree of subordination. Length‐based measures alone are therefore insufficient to capture the full linguistic demands placed on the speaker.

To address this limitation, other measures have been proposed. The Index of Syntactic Complexity (ISC, Szmrecsanyi 2004) estimates complexity based on the grammatical classes that make up a sentence and is well suited to French. However, it does not account for several relevant features such as passives, cleft constructions, or sentence modality (affirmative, negative, interrogative), which may also influence fluency. The Index of Productive Syntax (IPSyn; Scarborough 1990), used in Wagovich and Hall (2018), offers a different perspective by focusing on the presence or absence of specific syntactic structures, thereby disentangling the effects of utterance length and grammatical complexity. Although originally developed for child language, its conceptual framework can be adapted to adult data to capture a broader range of constructions possibly relevant to stuttering.

Other approaches to measuring syntactic complexity, such as clause‐based or dependency‐based metrics frequently used in computational linguistics (e.g., Kahane and Yan 2019), offer additional perspectives but were not retained in the present study. MLU, ISC, and IPSyn were selected because they provide complementary and well‐established metrics that are particularly suitable for French spontaneous speech, and they are relatively easy to use in a clinical setting.

Given the absence of a single gold standard for assessing syntactic complexity, the use of multiple indices shall provide a more comprehensive characterization of linguistic structure. In this study, MLU was used to assess utterance length, while ISC and IPSyn captured different dimensions of syntactic complexity. This approach allows us to examine whether syntactic complexity itself contributes to disfluency rates in AWS, or whether observed effects are primarily driven by utterance length (Kleinow and Smith 2000). We expect a correlation between ISC and the disfluency rate, since the literature shows a decrease in speech motor stability with longer and more complex sentences in AWS. Since IPSyn was shown to be linked with an increase in stuttered utterances in children, we also expect this link to persist in adulthood. Regarding MLU, we expect SLDs to be less sensitive to this measure, since it only reflects one single parameter.

## 4 Methods

### 4.1 Population

For this study, 24 adults were recruited, including 20 men and 4 women aged between 23 and 50 years (mean age: 29.96 years, SD (standard deviation): 8.14; see Table 1). Their level of education ranged from 2 to 8 years after the high‐school diploma (*baccalauréat*). The sample was divided into two groups: 12 PWS, including 10 men and 2 women (mean age: 30 years, SD: 8.46) and 12 gender‐, age‐ and education‐matched control participants (mean age: 29.92 years, SD: 8.17). Inclusion criteria were: 18 years or older, French as a native language, and, for the target group, persistent developmental stuttering. Exclusion criteria were: the presence of any language disorder other than stuttering and any uncorrected visual or auditory impairment. The average level of education amongst PWS was 4.5 years after the high‐school diploma (SD: 1.88), and among control participants it was 4.33 years (SD: 1.88).

**TABLE 1:** Participants. PWS: person who stutters, CS: control subject. F: female, M: male. Age is provided in years. Stuttering severity is provided for PWS only.

Participant	Gender	Age	Stuttering severity
PWS1	M	27	Moderate
PWS2	M	24	Moderate
PWS3	F	35	Mild
PWS4	F	23	Mild
PWS5	M	43	Mild
PWS6	M	27	Mild
PWS7	M	29	Mild
PWS8	M	23	Moderate
PWS9	M	26	Moderate
PWS10	M	26	Moderate
PWS11	M	27	Severe
PWS12	M	50	Severe
CS1	M	27	
CS2	M	24	
CS3	F	38	
CS4	F	23	
CS5	M	39	
CS6	M	27	
CS7	M	29	
CS8	M	24	
CS9	M	26	
CS10	M	27	
CS11	M	25	
CS12	M	50	

**FIGURE 1:**
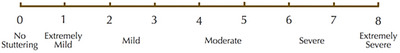
Stuttering Severity Scale, O'Brian and Cary, 2013.

For the PWS group, stuttering severity was assessed individually using a self‐report scale from the Camperdown program (O'Brian and Carey 2013, see Figure 1). Participants who rated their stuttering below 2 were excluded from the study. Based on these ratings, participants were classified into three groups: mild stutterers (5 PWS who rated their stuttering below 5), moderate stutterers (5 PWS who rated their stuttering at 5) and severe stutterers (2 PWS who rated their stuttering above 5).

### 4.2 Materials

The instructions were identical for all participants to avoid bias. They were displayed on the screen and read aloud by the experimenter. Participants were asked to describe the image shown on the screen as simply as possible, in a single sentence. A model was provided at the beginning of each image set (see below). For example, when the model was “The star is red.”, participants were expected to produce similar sentences such as “The table is yellow.” or “The computer is grey.”. The task was directed, as it required the use of specific syntactic constituents.

All sentences were based on images from the E.CO.S.SE (Epreuve de Compréhension Syntaxico‐Sémantique (Test of syntactic‐semantic comprehension), Lecocq 1998) speech therapy test. The sentences were selected and, in some cases, modified to fit the goals of the present study. Only declarative sentences were included (i.e., no negation or interrogatives), as in the stuttering study by Hollister et al. (2017). They varied in syntactic complexity to create different levels of linguistic load. The four sentence types were inspired by Usler and Walsh (2018), and their complexity levels were comparable to those used in the literature (e.g., Watson et al. 2011). Longer sentences were included to allow variation in coordination type and to introduce phonological variability, as certain sounds are known to elicit stuttering more frequently (Blomgren et al. 2012).

Sentences varied in length (through coordination) and syntactic complexity (through subordination). The slideshow thus contained three short, simple sentences with no subordination or coordination (Group 1), such as “The boy is reading”. This group was followed by three short, complex sentences with a subordinate clause (Group 2), such as “The shoe on the chair is blue”. The next nine sentences were simple and long, with coordination only. Group 3 included three sentences with two coordinated noun phrases (“The eraser that's green is between a pencil and a sharpener.”), Group 4 included three sentences with two coordinated adjectival phrases (“The box is big and white.”), and Group 5 included three sentences with two coordinated clauses (Group 5) (“The boy drinks and the girl eats.”). Finally, the last set of sentences consisted of nine long, complex sentences with both subordination and coordination. Group 6 included three sentences with a relative clause and two coordinated noun phrases (“The eraser that is green is between a pencil and a sharpener. “), Group 7 included three sentences with a relative clause with two coordinated noun phrases, each containing an adjective (”The cat is looking at the girl with the black hair and blue eyes.“), and Group 8 included three sentences with a relative clause with two coordinated clauses (Group 8) (”The boy with the yellow shoes is drinking and the girl is eating.”). Thus, each stimuli elicited one utterance produced by the participants.

The syntactic complexity of each sentence was calculated in two steps: an “expected” value (based on the sentence design) and an actual value (based on participant production). Three measures were used: the first measure was based on the ISC (Szmrecsanyi 2004), as follows: 2 x n(u, SUB) + 2 x n(u, WH) + n(u, VF) + n(u, NP), where n = number of occurrences in the utterance, u = utterance, SUB = subordinating conjunction, WH = relative pronoun, VF = verb and NP = nominal phrase. The second measure was the IPSyn, which takes into account the presence of specific structures (two points for ≥ 2 occurrences of the structure, one point for one occurrence or zero points for no occurrence). Finally, the last measure was the MLU, automatically calculated in CLAN (MacWhinney 2000) based on the number of morphemes in each utterance.

These three indices were selected to compare their sensitivity to different sentence types. MLU primarily reflects sentence length, making it particularly useful for distinguishing between short, simple sentences and long, simple sentences containing coordination. It is also widely used in stuttering research, especially in children (Richels et al. 2010; Zackheim and Conture 2003). In contrast, ISC and IPSyn provide more fine‐grained information on syntactic complexity, since they contain subordination. ISC focuses on sentence structure, whereas IPSyn emphasizes the presence of specific syntactic components.

### 4.3 Context and Environment

Due to the health situation during data collection in 2020, the study was conducted remotely using the Zoom videoconferencing platform. Each participant took part individually under the supervision of the experimenter. Both the participant and the experimenter were seated in quiet environments in front of their respective computers, each equipped with an integrated microphone and camera. The slideshow of images was displayed simultaneously to both parties via Zoom's screen‐sharing feature.

### 4.4 Procedure

After verifying the sound quality of the videoconference, the instructions were delivered orally. At the beginning of each session, an example was provided to ensure that the instructions were clearly understood. For each group of images, the first image was described by the experimenter as a model, and the participant was then asked to describe the next three images. Sessions lasted approximately five minutes for fluent participants, and between five and eight minutes for PWS, depending on stuttering severity. Participants were not informed of the purpose of the study prior to completing the task.

### 4.5 Data Processing

**FIGURE 2:**
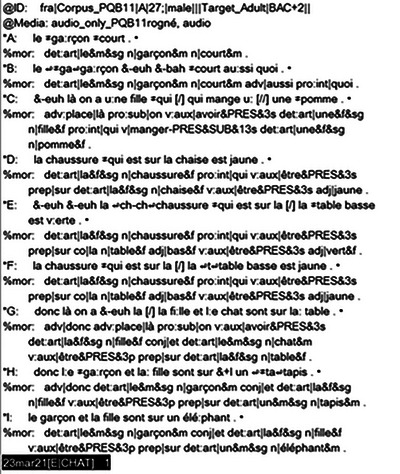
Screenshot of the CLAN interface and annotations.

The audio and video recordings were analyzed using CLAN software, which is part of the Child Language Data Exchange System (CHILDES) (MacWhinney 2000). CLAN was used to transcribe participants' utterances and annotate the disfluencies (Figure 2). Since each trial required exactly one utterance, utterances were segmented accordingly to trials, that is, one trial = one utterance. In all trials, utterances contained at least one verb, see Methods section for more information. It distinguishes between SLDs, such as blocks, repetitions, and prolongations—typically produced by PWS—and other disfluencies (ODs), including pauses, filled pauses, revisions, repetitions, and phonological fragments, which are produced by both PWS and AWNS. Transcriptions and annotations were completed manually by a trained Master's student in Speech and Language Pathology. All files were subsequently checked for accuracy by the third author. Disfluency proportions relative to the total number of words were calculated using the FLUCALC command in CLAN (see Fromm et al. 2024).

The choice of CLAN software was motivated by its reliability in producing consistent results and its potential applicability in clinical speech therapy practice. Two of the syntactic complexity indices used in this study (MLU and IPSyn) are integrated into CLAN, making it convenient for clinical use by allowing syntactic complexity to be calculated without the need for syntax trees or manual computations. Since IPSyn was originally developed for English, it was adapted for use in this study. ISC, although not integrated into CLAN, required only simple manual calculations, which also makes it suitable for clinical applications.

CLAN software (MacWhinney 2000) was also used for morphological segmentation of utterances via the “mor” module, a necessary step for calculating MLU.

For statistical analysis, non‐parametric tests were employed. The Mann‐Whitney test was used to compare two means, while the Kruskal‐Wallis test was applied to compare multiple means. When the Kruskal‐Wallis test was used, it was followed by Wilcoxon pairwise comparisons with corrections for multiple comparisons. Correlations were analyzed using the correlation coefficient (R), with degrees of freedom (df) and statistical significance indicated by the *p*‐value.

## 5 Results

The data collected enabled an analysis of the types of disfluencies produced by the two groups (PWS and AWNS), a comparison of the syntactic complexity scores according to the three indices (MLU, ISC and IPSyn), a comparison of the number of disfluencies produced by the two groups and finally an analysis of the number of disfluencies according to the syntactic complexity of the utterances. As a reminder, our hypothesis was that the number of disfluencies of PWS increases as the syntactic complexity of the utterances increases (in terms of IPSyn and ISC).

### 5.1 The Number of Disfluencies

#### 5.1.1 Total Number of Disfluencies

As shown in Table 2, PWS produced significantly more SLDs and ODs per utterance at all complexity levels (mean: 1.024, SD: 1.556) than AWNS (mean: 0.41, SD: 0.682). The results show a significant effect of the total number of disfluencies according to population (*p* < 0.000). Severe stutterers tend to produce more disfluencies (mean: 3.208, SD: 2.042) than mild (mean: 0.617, SD: 0.909) or moderate stutterers (mean: 0.083, SD: 0.282).

**TABLE 2:** Total number of disfluencies per group and according to stuttering severity.

	AWNS	PWS	Mild	Moderate	Severe
Mean total number of disfluencies	0.41	1.024	0.617	0.083	3.208
Standard deviation	0.682	1.556	0.909	0.282	2.042
*p*‐value	0.000				

#### 5.1.2 SLDs

As shown in Table 3a, PWS produced significantly more SLDs (mean: 0.684, SD: 1.283) than AWNS (mean: 0.097, SD: 0.297). The results show a significant effect of population on the number of SLDs (*p* < 0.000). In terms of stuttering severity, severe stutterers also tend to produce more SLDs (mean: 2.75, SD: 1.78) than mild (mean: 0.208, SD: 0.428) or moderate stutterers (mean: 0.042, SD: 0.204).

**TABLE 3a:** Number of SLDs per utterance per group.

	AWNS	PWS overall	Mild	Moderate	Severe
Mean number of SLDs	0.097	0.684	0.208	0.042	2.75
Standard deviation	0.297	1.283	0.428	0.204	1.78
*p*‐value	0.000				

#### 5.1.3 ODs

Statistical tests revealed no difference (*p* = 0.765) in the number of ODs produced by AWNS (mean: 0.312, SD: 0.59) and by PWS (0.34, SD: 0.7, Table 3b). With regard to the degree of severity of stuttering, severe stutterers had a slight tendency to produce more ODs (mean: 0.458, SD: 0.771) than mild (mean: 0.408, SD: 0.716) or moderate stutterers (mean: 0.042, SD: 0.204).

**TABLE 3b:** Number of ODs per utterance per fluency group.

	AWNS	PWS	Mild	Moderate	Severe
Mean number of ODs	0.312	0.34	0.408	0.042	0.458
Standard deviation	0.59	0.7	0.716	0.204	0.771
*p*‐value	0.765				

### 5.2 Type of Disfluency

#### 5.2.1 Type of Disfluencies in AWS

The analysis of SLDs in Table 4 shows that PWS produced an average of 0.267 block (SD: 0.679), 0.208 prolongation (SD: 0.533), and 0.125 sound or syllable repetition (SD: 0.371) per utterance. Among ODs, PWS produced an average of 0.17 filled pause (SD: 0.467), 0.083 word repetition (SD: 0.289), 0.069 pause (SD: 0.268), 0.049 sentence revision (SD: 0.215), as many sentence repetitions as word revisions (average: 0.021, SD: 0.143) and 0.01 phonological fragments (SD: 0.102). The results show a significant effect of disfluency type in PWS (*p* = 0.002).

**TABLE 4:** Mean number and type of disfluencies per utterance by fluency group.

	PWS		AWNS	
	Mean	SD	Mean	SD
Prolongations	0.208	0.533	0.087	0.282
Blocks	0.267	0.679	0	0
Repetitions (sounds, syllables)	0.125	0.371	0.003	0.059
**Total SLDs**	**0.684**	**1.283**	**0.097**	**0.297**
Repetition (words)	0.083	0.289	0.007	0.083
Phonological fragments	0.01	0.102	0.003	0.059
Repetition (sentences)	0.021	0.143	0.007	5.831
Revision (words)	0.021	0.143	0.003	0.059
Revision (sentences)	0.049	0.215	0.017	0.131
Pauses (silent)	0.069	0.268	0.083	0.289
Pauses (filled)	0.17	0.467	0.187	0.441
**Total ODs**	0.34	0.7	0.312	0.59
*p*‐value	0.002		0.000	

#### 5.2.2 Type of Disfluency in Adults Who Do Not Stutter

Analysis of the SLDs shown in Table 4 shows that AWNS produced an average of 0.087 prolongation (SD: 0.282), almost no repetition of sounds or syllables (mean: 0.003, SD: 0.059) and no block. Among ODs, they produced an average of 0.187 filled pause (SD: 0.441), 0.083 pause (SD: 0.289), 0.017 sentence revision (SD: 0.131), 0.007 word repetition (SD: 0.083) and as many sentence repetitions (mean: 0.007, SD: 5.831) and almost no phonological fragment or word revision (mean: 0.003, SD: 0.059). The results show a significant effect of disfluency type in AWNS (*p* < 0.000).

### 5.3 Calculating Syntactic Complexity According to the Three Indices

The aim of this subsection is to verify the relevance of the eight syntactic complexity groups and the sensitivity of the measures used to calculate this complexity.

As Table 5 shows, the complexity groups are well differentiated in terms of syntactic complexity, whether using MLU (*p* = 0.000), ISC (*p* = 0.000) or IPSyn (*p* = 0.000). Groups 1 and 4 had the lowest syntactic complexities, while groups 7 and 8 had the highest. The remaining groups (2, 3, 5 and 6) had syntactic complexity scores in between. Wilcoxon pairwise comparison tests showed significant differences for all groups and all measures except groups 2 and 3 for MLU, groups 7 and 8 for ISC and groups 6 and 8 for IPSyn.

**TABLE 5:** Expected and actual values of syntactic complexity by measure of syntactic complexity (in columns), by fluency group (PWS: people who stutter, AWNS: people who don't stutter), and by sentence group (in rows, from G1 to G8). M = mean, SD = standard deviation.

	MLU: expected		MLU: PWS		MLU: AWNS		ISC: expected		ISC: PWS		ISC: AWNS		IPSyn: expected		IPSyn: PWS		IPSyn: AWNS	
	M	SD	M	SD	M	SD	M	SD	M	SD	M	SD	M	SD	M	SD	M	SD
G1	3.333	1.592	4.083	0.577	3.778	1.198	2	0	2.333	0.894	2.194	0.401	7	0	8.694	3.012	8.472	2.793
G2	9	0.232	9.056	0	9.111	0.465	8	0	8	0	8	0	24	0	24.056	0.232	24.056	0.232
G3	9	1.298	9.5	0	9	1.298	4	0	4.028	0.167	3.944	0.232	18	0	18.139	0.593	18.028	0.377
G4	6	0	6	0	6.056	0	2	0	2	0	2	0	12	0	12	0	12.056	0.333
G5	7.667	1.069	8	0.577	7.778	0.721	4	0	4.194	0.467	4.056	0.232	17	0	17.444	1.054	17.083	0.5
G6	12.333	0.828	12.667	0.577	12.333	0.828	9	0	9	0	8.583	1.401	25	0	24.722	1.485	24.833	0.561
G7	18	1.183	18.528	0	17.972	0.167	10	0	9.833	0.697	10	0	27	0	27.028	1.23	26.917	0.368
G8	13	1.082	13.528	1	13.694	1.879	10	0	9.889	1.282	10.278	0.849	25	0	25	1.621	25.083	0.806
M	9.792	4.567	10.17	4.601	9.965	4.53	6.125	3.482	6.16	3.364	6.132	3.45	19.375	7.15	19.635	6.696	19.566	6.765

#### 5.3.1 The Difference Between Expected and Actual Syntactic Complexity

##### The Difference for AWS

Analysis of the scores shown in Table 5 reveals a slight upward trend between expected and actual scores for the overall averages of the three indices. The MLU went from an expected mean of 9.792 (SD: 4.567) to an actual mean of 10.17 (SD: 4.601), the ISC from an expected mean of 6.125 (SD: 3.482) to an actual mean of 6.16 (SD: 3.364) and the IPSyn from an expected mean of 19.375 (SD: 7.15) to an actual mean of 19.635 (SD: 6.696).

However, these differences were not significant (*p* = 0.674 for MLU, *p* = 0.958 for ISC and *p* = 0.916 for IPSyn). On the other hand, a slight downward trend was observed for complexity groups 7 and 8 with the ISC and for group 6 with the IPSyn. The expected score corresponded exactly to the actual score for several statement groups: group 4 with the MLU, groups 2, 4 and 6 with the ISC and groups 4 and 8 with the IPSyn. This absence of significant differences confirms compliance with the instructions and expected complexities before proceeding with the following analyses.

##### The Difference for AWNS

Analysis of the scores for the three indices shown in Table 5 reveals a slight upward trend between expected and actual scores for the overall averages of the three indices. The MLU went from an expected mean of 9.792 (SD: 4.567) to an actual mean of 9.965 (SD: 4.53), the ISC from an expected mean of 6.125 (SD: 3.482) to an actual mean of 6.132 (SD: 3.45) and the IPSyn from an expected mean of 19.375 (SD: 7.15) to an actual mean of 19.566 (SD: 6.765).

This increase mainly concerned three groups (1, 5 and 8). For the other groups (2, 3, 4, 6 and 7), the score difference varied according to index.

The differences were not significant (MLU: *p* = 0.833; ISC: *p* = 0.916; IPSyn: *p* = 0.875). Again, this lack of significant difference confirms that the instructions and expected complexities have been respected.

### 5.4 Number of Disfluencies Per Syntactic Complexity

#### 5.4.1 Number of Disfluencies Produced by PWS Per Syntactic Complexity

Analysis of the results in Table 6 shows that the number of disfluencies produced by PWS increased significantly as a function of the value of MLU (*p* = 0.000, R = 0.209, dl = 286), ISC (*p* = 0.033, R = 0.126, dl = 286), and IPSyn (*p* = 0.015, R = 0.143, dl = 286). However, correlation coefficients show weak to very weak relationship.

**TABLE 6:** Mean number of disfluencies (ODs and SLDs) per sentence group and fluency group. PWS = persons who stutter, AWNS = adults who do not stutter, M = mean, SD = standard deviation.

	PWS		AWNS	
	M	SD		
G1	1	1.707	0.417	0.649
G2	0.806	1.39	0.333	0.736
G3	1.5	1.797	0.556	0.809
G4	1.632	3.126	0.278	0.659
G5	0.639	1.268	0.361	0.543
G6	1.361	1.791	0.528	0.845
G7	1.194	1.369	0.417	0.604
G8	1.167	1.765	0.389	0.599

MLU explains the number of total disfluencies produced by PWS significantly better than ISC (*p* = 0.029) and IPSyn (*p* = 0.031). On the other hand, ISC and IPSyn are identical in explaining the number of total disfluencies in people who stutter (*p* = 0.73).

#### 5.4.2 The Number of Disfluencies Produced by AWNS According to Syntactic Complexity

Analysis of the results in Table 6 shows that the number of disfluencies in AWNS did not increase significantly as a function of the value of MLU (*p* = 0.252, R = 0.068, dl = 286), ISC (*p* = 0.453, R = 0.044, dl = 288) and IPSyn (*p* = 0.429, R = 0.047, dl = 286). When compared, no index explained the number of total disfluencies produced by AWNS significantly better than others: neither MLU compared to ISC (*p* = 0.309) or IPSyn (*p* = 0.537), nor ISC compared to IPSyn (*p* = 0.573).

The correlation coefficient (R) being too low for these three calculation measures (for MLU : R = 0.068, *p* = 0.25; for ISC: R = 0.044, *p* = 0.45; for IPSyn: R = 0.047, *p* = 0.43), it does not allow to determine whether one of them is more relevant.

## 6 Discussion and Conclusion

Stuttering is known to be influenced by a range of factors, including language and syntax. While the relationship between syntactic complexity and stuttering has been well documented in children, its role in AWS remains unclear. In this study, we investigated the relationship between syntactic complexity—measured by three different indices—and SLDs and ODs in French‐speaking AWS and who do not stutter (AWNS). We expected a positive association between syntactic complexity and disfluency rates in AWS but not in AWNS, and we further hypothesized that indices capturing structural properties (i.e., ISC or IPSyn) would better reflect this relationship than sentence length alone (i.e., MLU). To test these hypotheses, 12 AWS and 12 AWNS performed an image description task involving groups of sentences with varying syntactic complexity. Disfluencies and syntactic complexity of each utterance were analyzed.

As anticipated, AWS produced significantly more SLDs and slightly more ODs than AWNS. This result was expected, since SLDs are rare in the speech of AWNS.

Regarding disfluency types, PWS produced significantly more SLDs than ODs. Their disfluencies primarily consisted of blocks, followed by prolongations, filled pauses and repetitions of sounds or syllables. Filled pauses were frequent in both AWS and AWNS.

Unlike Manning (2009), who suggested that adults may rely more on syntactic (as opposed to phonological) disfluencies such as revisions, our results did not show a higher proportion of revisions relative to repetitions or prolongations. Manning (2009) posited that syntactic disfluencies might be a strategy to reformulate an utterance in order to avoid stuttering. While our findings do not support this explanation, it remains possible that AWS adjust their production to avoid overly complex sentences, thereby reducing the risk of stuttering. Such adaptive strategies may not always manifest as overt revisions but may instead operate earlier in the formulation process, influencing utterance structure. To investigate this, we first examined whether the three selected syntactic complexity measures (MLU, ISC, and IPSyn) aligned with the expected sentence groups based on syntactic complexity.

The results show that all three measures reliably distinguished groups of sentences according to their complexity, though the gradation across groups was not entirely linear. For example, Group 1 has the least complex syntax, while Groups 7 and 8 had the most complex syntax. Group 4, expected to rank fourth in complexity, was instead categorized as the second least complex group. This discrepancy may reflect the subtle differences in syntactic complexity among groups. Groups 3, 4 and 5, for instance, all featured coordination without subordination, but varied in the types of coordination used (Riegel et al. 2009): coordination of two noun phrases for Group 3, two adjectival phrases for Group 4, and two propositions for Group 5. Among these, adjectival phrase coordination (without subordination) appeared the least complex.

Importantly, no significant differences emerged between expected and produced syntactic complexity for MLU, ISC, or IPSyn, indicating that participants generally followed the expected sentence structure and thus validating the protocol used. Participants, on average, produced slightly more complex utterances than expected. AWS, in particular, showed a slight tendency to deviate from the model, which could reflect strategic adjustments to ease speech production, such as the use of alternative forms or lexical fillers (Junuzovic‐Zunic and Ibrahimagic 2013). While this tendency was not statistically robust, it is consistent with prior accounts of anticipatory avoidance or facilitation strategies in stuttering.

When examining the relationship between syntactic complexity and disfluency, we found no significant association in AWNS, but a positive (though weak) correlation in AWS, indicating that utterances with higher complexity scores were more likely to contain disfluencies. Although the effect was modest, it is consistent with studies showing that longer or syntactically more demanding utterances increase disfluency rates in children (Buhr and Zebrowski 2009; Usler and Walsh 2018; Wagovich and Hall 2018). These findings suggest that the influence of syntactic load persists into adulthood, though it may be one of several interacting factors contributing to stuttering.

Contrary to what might be expected if syntactic mastery compensated for stuttering vulnerability (Martinot 2013), AWS did not produce fewer disfluencies on more complex utterances. This suggests that age‐related language development does not fully mitigate fluency breakdowns. Rather, syntactic complexity may place additional demands on linguistic planning and motor programming (Kleinow and Smith 2000; Weber‐Fox and Hampton 2008), increasing the likelihood of disfluency in individuals with reduced speech motor stability. Loucks and De Nil (2006) have also shown that differences in proprioceptive feedback may disrupt sensory‐motor integration in PWS, which could amplify the impact of linguistic load.

Overall, these results point to a trend, rather than a strong effect, whereby increased syntactic complexity is associated with a higher likelihood of disfluency in PWS. This effect should be interpreted with caution, given the relatively small sample size and the moderate strength of the association. Syntactic complexity likely constitutes one component of a broader set of factors including prosodic, lexical, and motor constraints that interact in shaping fluency. Future studies with larger samples, finer‐grained syntactic manipulations, and experimental control over linguistic variables will be needed to clarify the scope and strength of this relationship.

## 7 Limitations and Future Directions

This study has several limitations that should be considered when interpreting the results. First, the small sample size limits the generalizability of our findings and may have contributed to the modest strength of the observed effects. Larger and more balanced samples would increase statistical power and may reveal more robust associations between syntactic complexity and disfluency. In addition, the distribution of stuttering severity levels was uneven, with fewer participants in the severe group compared to the mild and moderate groups, which may have reduced sensitivity to severity‐related differences.

Second, the directed task methodology may have influenced the results. The directed image description task likely constrained both sentence form and content, potentially leading to fewer disfluencies than might occur in most naturalistic contexts. Furthermore, the task focused only on coordination and subordination, excluding other syntactic structures (e.g., negation, interrogatives, passive voice) that contribute to syntactic complexity in French. Incorporating a broader range of sentence types and additional syntactic indices would allow for a more comprehensive assessment of linguistic load.

Third, the methodological choice of a controlled task has both strengths and drawbacks. While spontaneous speech could provide a richer and more ecologically valid picture of syntactic complexity, it also gives speakers the opportunity to anticipate linguistic demands and adjust their utterances to avoid stuttering. Conversely, reading tasks, although associated with lower disfluency rates in PWS, may allow for tighter control over syntactic variables and more direct comparison between expected and produced structures. A combination of spontaneous and controlled tasks may therefore provide a better complete view of the interaction between syntactic complexity and stuttering.

Finally, this study focused exclusively on syntactic factors, but stuttering is influenced by multiple linguistic and motor dimensions. Phonological properties, such as sound type and position within words or sentences, are known to modulate disfluency likelihood. Future studies should integrate phonological and syntactic variables, potentially through multifactorial models, to better capture the interplay between linguistic planning and speech motor execution in AWS.

### 7.1 Assessing the Impact on the Speech‐Language Pathology Profession

This study provides new elements to be taken into account in clinical speech therapy practice and research, particularly for the development of therapy protocols. Although most individuals who consult a speech‐language pathologist for stuttering are pre‐school and school‐age children, adolescents and AWS can also benefit from intervention. According to the recommendations of the Lidcombe program (Onslow et al. 2015), based on the Demands and Capacities model (Starkweather and Gottwald 1990) for preschoolers, parents of children who stutter are encouraged to adapt their language to reduce external (environmental) demands, so that they better match the child's internal abilities. This involves simplifying language, especially at the syntactic level, by using shorter, less complex sentences. If the present findings are any indication, similar strategies could also be relevant in the environment of AWS, since syntactic complexity appears to play a role in their fluency.

Speech‐language pathologists could also work to strengthen the linguistic abilities of PWS, helping them better meet external linguistic demands and thereby potentially reduce disfluencies. A stronger command of syntax, in particular, could provide more stable linguistic foundations and facilitate fluency. Since adult stuttering therapy typically involves both speech restructuring (such as the Camperdown program, O'Brian and Carey 2013), and work on awareness and acceptance of the disorder (Blomgren 2013), improving syntactic skills would possibly may enhance speaker's understanding of their own language and support greater control over it.

Finally, this study showed that MLU is the measure that best explained the number of disfluencies produced. Because it can be calculated automatically in French, MLU represents a reliable and accessible tool that can be easily integrated into clinical practice.

## 8 Conclusion

Numerous studies have investigated the origins and components of stuttering. Among the factors explored, syntax appears to play a meaningful role in the language production of people who stutter. Beyond phonological and lexical aspects, sentence length and complexity have often been associated with stuttering: more complex sentences tend to be linked to a higher likelihood of disfluency in PWS (Wells 1979; Kleinow and Smith 2000). Building on findings in children who stutter, the present study sought to determine whether a similar relationship exists in AWS.

Previous research has relied on a range of tools to estimate syntactic complexity, leading to methodological inconsistencies. Because sentence length alone does not always capture complexity (a long sentence with coordination may be easier to produce than a short sentence with subordination), this study compared the widely used MLU with two indices incorporating subordination: the ISC and the IPSyn.

The results suggest that PWS produce more disfluencies as syntactic complexity increases, although the observed effect was modest. Among the three indices tested, MLU emerged as the measure most strongly associated with disfluency rates. While MLU focuses on length rather than structure, its performance in this study indicates that it remains a practical and sufficiently sensitive tool for capturing relevant variability in utterance production. Its automatic calculation in French through software such as CLAN also makes it particularly suitable for clinical and research settings.

Overall, this study adds to growing evidence that syntactic complexity is one of several factors shaping stuttering patterns in adults. These findings also resonate with the principles of the Demands and Capacities Model (Starkweather and Gottwald 1990), which underpins clinical programs such as the Lidcombe program (Onslow et al. 2015). Extending such frameworks to AWS could help clinicians better balance external linguistic demands with individual capacities. However, given the modest strength of the observed effects, further research using larger and more diverse samples will be needed to confirm and refine these conclusions.

## Funding

This study was supported by ANR (Agence Nationale de la Recherche) grant no. ANR‐18‐CE36‐0008. The authors certify that they have no affiliation with or involvement in any organization or entity with any financial interest, or non‐financial interest in the subject matter or materials discussed in this manuscript.

## Consent

All participants provided informed consent for participation in the study and for the use of their anonymized data for research and publication purposes. No identifiable personal information is reported.

## Conflicts of Interest

The authors declare that they have no known competing financial interests or personal relationships that could have appeared to influence the work reported in this paper.

## Data Availability

The data that support the findings of this study are available on request from the corresponding author. The data are not publicly available due to privacy or ethical restrictions.
